# Type I Interferon Potentiates IgA Immunity to Respiratory Syncytial Virus Infection During Infancy

**DOI:** 10.1038/s41598-018-29456-w

**Published:** 2018-07-23

**Authors:** Diego R. Hijano, David T. Siefker, Bishwas Shrestha, Sridhar Jaligama, Luan D. Vu, Heather Tillman, David Finkelstein, Jordy Saravia, Dahui You, Stephania A. Cormier

**Affiliations:** 1 Department of Infectious Diseases, St. Jude Children’s Research Hospital, Memphis, Tennessee USA; 2 Department of Biological Sciences, Louisiana State University, Baton Rouge, Louisiana USA; 3 Department of Pediatrics, University of Tennessee Health Science Center, Memphis, Tennessee USA; 4 Department of Pathology, St. Jude Children’s Research Hospital, Memphis, Tennessee USA; 5 Department of Computational Biology, St. Jude Children’s Research Hospital, Memphis, Tennessee USA

## Abstract

Respiratory syncytial virus (RSV) infection is the most frequent cause of hospitalization in infants and young children worldwide. Although mucosal RSV vaccines can reduce RSV disease burden, little is known about mucosal immune response capabilities in children. Neonatal or adult mice were infected with RSV; a subset of neonatal mice received interferon alpha (IFN-α) (intranasal) prior to RSV infection. B cells, B cell activating factor (BAFF) and IgA were measured by flow cytometry. RSV specific IgA was measured in nasal washes. Nasal associated lymphoid tissue (NALT) and lungs were stained for BAFF and IgA. Herein, we show in a mouse model of RSV infection that IFN-α plays a dual role as an antiviral and immune modulator and age-related differences in IgA production upon RSV infection can be overcome by IFN-α administration. IFN-α administration before RSV infection in neonatal mice increased RSV-specific IgA production in the nasal mucosa and induced expression of the B-cell activating factor BAFF in NALT. These findings are important, as mucosal antibodies at the infection site, and not serum antibodies, have been shown to protect human adults from experimental RSV infection.

## Introduction

Respiratory syncytial virus (RSV) is the most common cause of lower respiratory tract infections in infants and young children worldwide and is associated with high rates of hospitalization^1^. Globally, RSV infections occur in approximately 64 million people and lead to 160,000 infant deaths each year^2^. In the United States, RSV infections are annually associated with 85,000–144,000 infant hospitalizations^2^. Infants younger than 3 months are at highest risk for RSV-related hospitalizations, most of which occur during the first year of life^3,4^. Age is an important risk factor for severe RSV in children^5,6^. Increased severity of RSV infections in young infants can be due to structural immaturity of airways^7^ and/or immaturity of the immune system^8–10^.

There are well-defined differences between innate and adaptive immune responses of infants and adults^11,12^. Neonatal mouse models of RSV infection have revealed the key role of age-dependent differences in RSV pathogenesis^13–16^, of which those related to type I interferon (IFN) production are of special interest. RSV is a poor inducer of IFN^17–19^, and nonstructural proteins NS1 and NS2 can suppress IFN production^20,21^. Neonatal mice cannot induce a robust type I IFN immune response, which is consistent with findings from human studies^22,23^. However, RSV infection–induced IFN-alpha (IFN-α) production is significantly higher in adult mice than in neonatal mice^22,23^, suggesting that RSV can suppress type I IFN production but responses of neonatal and adult mice are distinct. Furthermore, engagement of type I IFN pathways is associated with T helper type 1 (Th1)–biased immune responses and less airway hyper-reactivity after RSV reinfection in mice^24^. Age-based differences in type I IFN production and pathways involving B-cell proliferation and maturation have been reported in very young humans^25^.

The mucosal immune system is an integrated network of tissues, cells, and effector molecules that protects the host from infections and environmental insults at mucosal surfaces^26^. Mucosal surfaces are immunologically unique, acting both as a barrier and as the primary interface between the host and physical environment from which the pathogen arrives^26,27^. Mucosal antibodies against RSV, especially IgA, mediate protection against experimental RSV infection in adults^28,29^. Vissers *et al*. proposed that mucosal IgG levels have higher correlation with disease protection than do serum IgG levels^30^. Despite the heightened interest in developing mucosal vaccines against various types of microbial pathogens for very young children^31–33^, little is known about the mucosal immune response capabilities of this patient population.

In this study, we use adult and neonatal mice infected with RSV to reveal the critical role of IgA at the nasal mucosa and an age-dependent deficit in IgA production. We show that this deficit in neonatal mice is due to decreased B-cell activation and can be ameliorated by IFN-α supplementation to the nasal mucosa before RSV infection.

## Results

### IFN-α decreases viral load and attenuates the immune response

**Figure 1:**
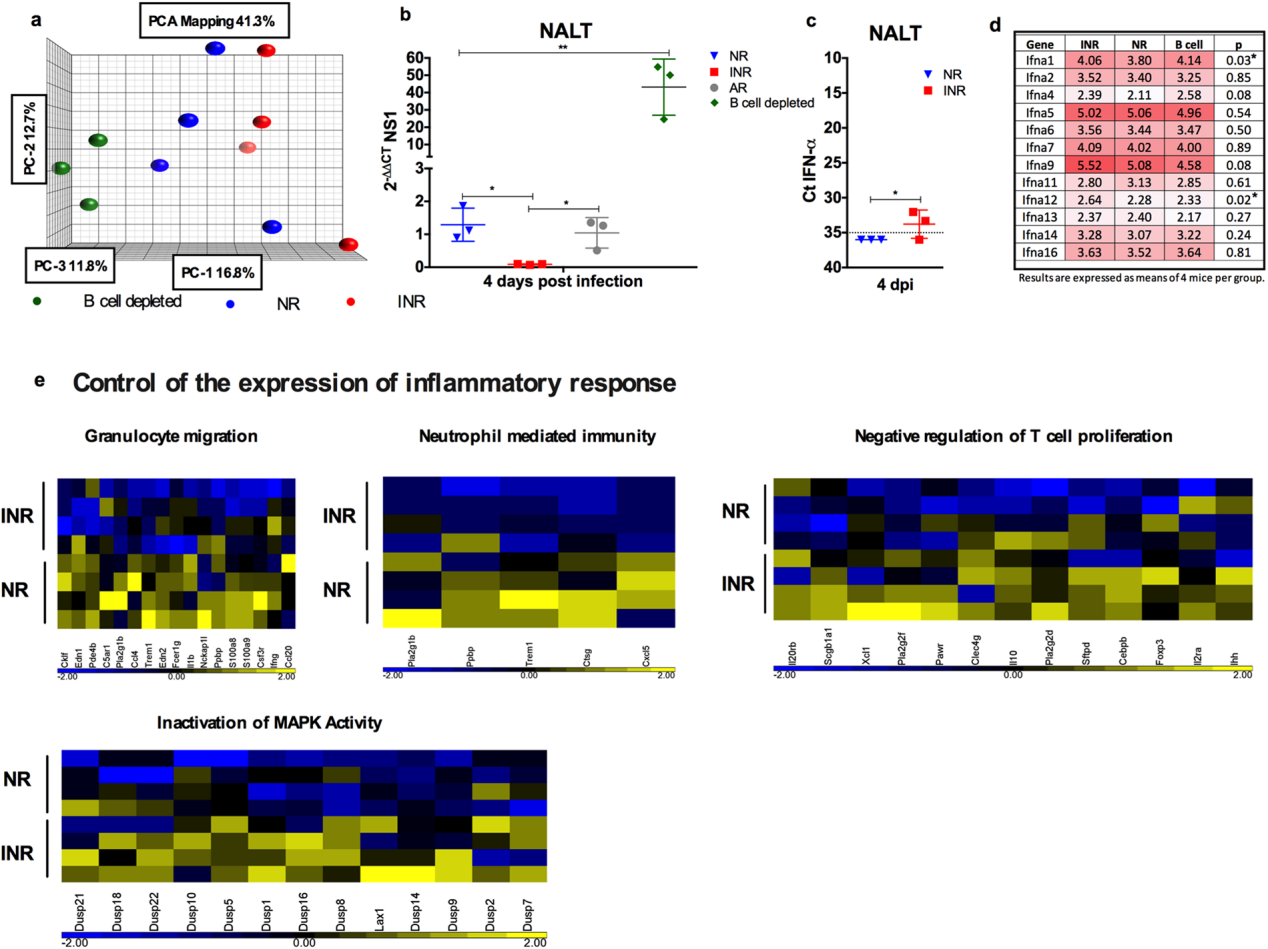
IFN-α decreases viral load and modulates gene expression upon RSV infection. (**a**) Principal component analysis of nasal-associated lymphoid tissue (NALT) from RSV-infected neonatal mice receiving IFN-α (INR/red), placebo (NR/blue), or anti CD-20 (green) 16 h before infection. (**b**) *NS1* gene expression in NALT from RSV-infected adult (gray) and neonatal mice receiving IFN-α (IRN/red), placebo (NR/blue), or anti CD-20 (green) 16 h before infection. (**c**) IFN-α 4 gene expression in NALT from RSV-infected neonatal mice receiving IFN-α (INR/red) or placebo (NR/blue) 16 h before infection. (**d**) IFN-α gene expression from RSV-infected neonatal mice receiving IFN-α (INR), placebo (NR), or anti CD-20 16 h before infection. (**e**) Heatmap analysis of data corresponding to fold change in the expression of indicated markers in NALT from RSV-infected neonatal mice receiving IFN-α (INR/red) or placebo (NR/blue) 16 h before infection. n = 3–5; N = 2 *P < 0.05, **P < 0.01.

Our previous work demonstrated that adult mice have higher type I IFN responses relative to neonates after being infected with RSV. Further, administration of IFN-α induced protection against RSV infection in neonates (i.e. decreased Th2-biased immunopathogenesis and attenuated airways hyperreactivity, pulmonary inflammation, and mucus hyperproduction)^23^. To further explore the protective effect of IFN-α in the immune response to RSV, host gene expression analysis on NALT and lung homogenates was performed at 4 days post-infection (dpi), when RSV viral load peaks in the respiratory tract^15^. Principal component analysis (PCA) showed significant differences in gene expression across the groups in both NALT and lungs (Fig. 1a and Supplementary Fig. 2e). PCA shows that first component defines a gradient of expression that distinguishes NR, INR, and B-cell depleted mice (Fig. 1a). RSV viral load in NALT was significantly lower in neonatal mice receiving IFN-α prior to RSV infection than in neonatal mice not receiving IFN-α or adult mice infected with RSV (Fig. 1b). Interestingly, RSV viral load in the lungs exhibited a similar pattern (i.e. significantly lower virus in neonatal mice receiving IFN-α prior to RSV infection than in neonatal mice not receiving IFN-α Supplementary Fig. 2f). A third group of neonatal mice depleted of B cells had significantly higher viral load compared to all other groups. Interestingly, IFN-α administration induced the expression of its homolog gene in NALT, suggesting host modulation of gene expression (Fig. 1c). Furthermore, IFN-α suppressed the immune response, as evidenced by decreased expression of genes involved in granulocyte migration and neutrophil-mediated immunity and upregulation of genes controlling the inflammatory response, such as those involved in the negative regulation of T-cell proliferation and MAPK activity (Fig. 1e and Supplementary Fig. 2c). To determine whether these results are due to IFN administration prior to infection, a result of an attenuated infection or a combination of both, we repeated the experiment comparing mice that received IFN-α with a group on neonatal mice that received palivizumab prior to RSV infection. There were over 7000 genes that are statistically significantly different between the groups (Supplementary Fig. 2d). Although some of the genes involved in T cell regulation and inactivation of MAP K were also expressed and some overexpressed in the group treated with palivizumab (Supplementary Table 1), many of the genes involved in granulocyte migration and neutrophil mediated immunity were further overexpressed in those treated with palivizumab **(**Supplementary Table 2). Consistent with previous data published by our group, upon reinfection, neonatal mice receiving IFN-α had significantly lower inflammation in their lower respiratory tract than did age-matched control mice, suggesting that effects of IFN-α on the immune response against RSV go beyond primary infection (Supplemenatry Fig. 3). Following administration of IFN-α in neonatal mice, we observed decreases in RSV viral load and increases in IFN-α gene expression (Fig. 1b,c). We analyzed gene expression data from mice pretreated with IFN-α and mice pretreated with palivizumab (Supplementary Table 3). Both interventions are known to reduce viral load and attenuate infection. Mice pre-treated with palivizumab exhibited strong overexpression of several IFN-α genes when compared to those who received IFN-α prior to RSV infection (Supplementary Table 3). Taken together, these findings suggest that upon RSV infection, IFN-α plays a dual role as an antiviral and immune modulator.

### IFN-α increases B cells and B-cell activation

**Figure 2:**
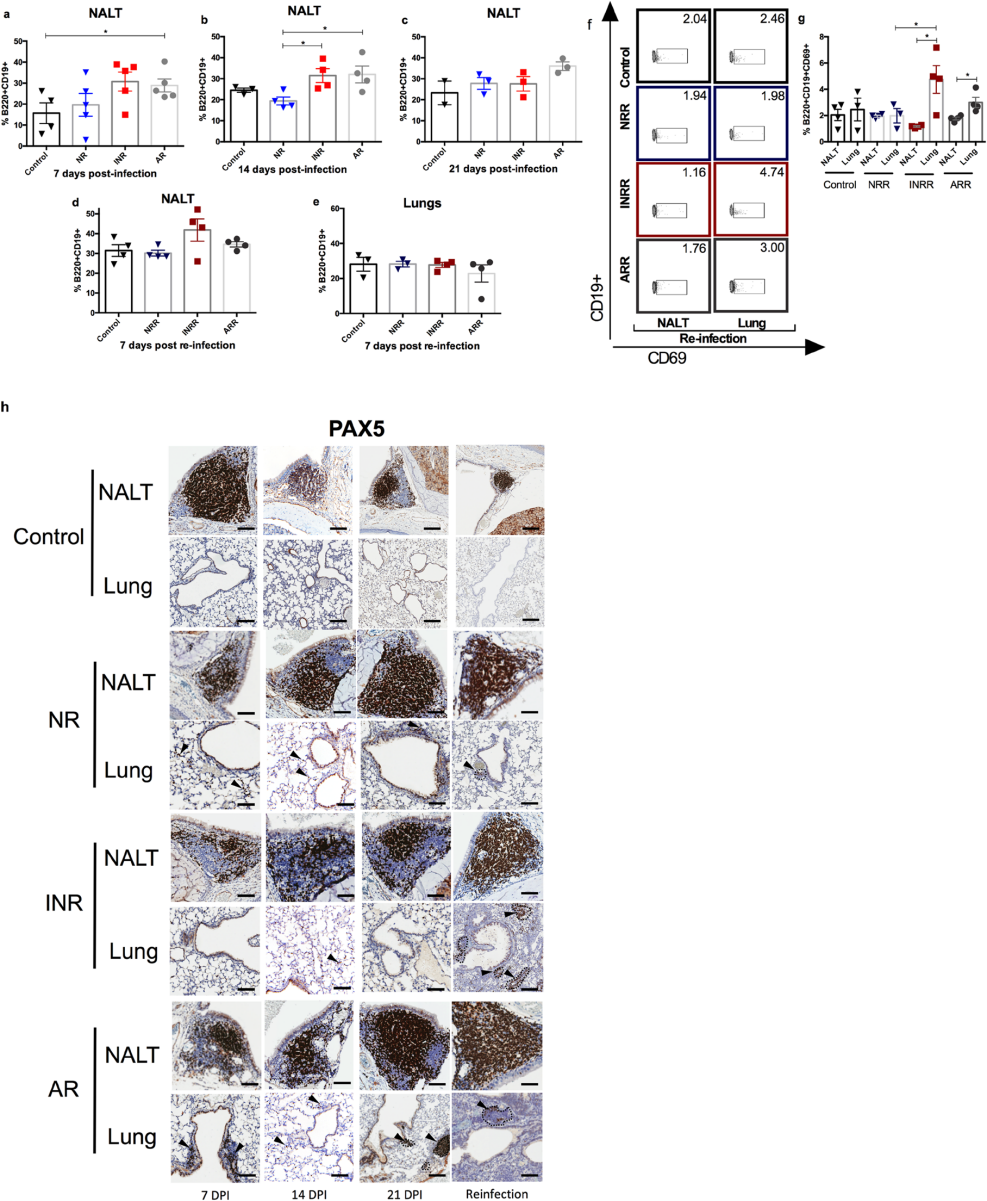
IFN-α administration before RSV infection correlates with increased B-cell lymphopoiesis in the respiratory tract after RSV infection *in vivo*. (**a**–**d**) Percentage of B220+CD19+ lymphocytes in NALT from RSV-infected adult (AR) and neonatal mice receiving IFN-α (INR) or placebo (NR) 16 h before RSV infection. Controls were inoculated with media. (**a**) 7 dpi; (**b**) 14 dpi; and (**c**) 21 dpi; (**d**,**e**) B cells in NALT and lungs upon secondary infection with RSV. (**d**) Percentage of B220^+^CD19^+^ lymphocytes in NALT from RSV-infected adult (ARR) and neonatal mice receiving IFN-α (INRR) or placebo (NRR) 16 h before RSV infection 7 days post-secondary infection. Controls were inoculated with media. (**e**) Percentage of B220^+^CD19^+^ lymphocytes in lungs from RSV-infected adult (ARR) and neonatal mice receiving IFN-α (INRR) or placebo (NRR) 16 h before RSV infection and 7 days post-secondary infection. Controls were inoculated with media. (**f–g**) B-cell activation in NALT and lungs from RSV-infected adult (ARR) and neonatal mice receiving IFN-α (INRR) or placebo (NRR) 16 h before RSV infection and reinfected 4 weeks after primary infection. (**f**) Representative flow cytometry panels of B220^+^CD19^+^CD69^+^ cells; (**g**) Percentage of B220^+^CD19^+^CD69^+^ lymphocytes in NALT and lungs. (**h**) IHC analysis demonstrating expression of PAX5 protein in NALT and lungs of RSV-challenged groups and control mice at 7, 14, and, 21 days post infection, as well as, after reinfection. Magnification 200×; scale bar = 100 μm. n = 3–5; N = 2 *P < 0.05, **P < 0.01.

To determine the role of B cells upon RSV infection, flow cytometry was performed on NALT to determine the percentage of CD19^+^B220^+^ lymphocytes. There was no difference in the percentage of CD19^+^B220^+^ lymphocytes among groups at 7 or 21 dpi (Fig. 2a,c). However, at 14 dpi, neonatal mice receiving IFN-α or adult mice had a significantly higher percentage of CD19^+^B220^+^ lymphocytes than did neonatal mice infected with RSV or control (Fig. 2b). There were no differences at 7 dpi after reinfection in NALT or lungs (Fig. 2d,e). Activated B cells, as measured by the percentage of B220^+^CD19^+^CD69^+^ lymphocytes, were higher in lungs of reinfected adult mice than of reinfected neonatal mice. Interestingly, the highest increase in activated B cells occurred in lungs of neonatal mice receiving IFN-α, suggesting a role for IFN-α in B-cell activation upon reinfection with RSV (Fig. 2f,g).

NALT was identified in H&E–stained sections within the lamina propria at the level of nasopharyngeal ducts, with variable formation also observed around the nasopharynx across different time points after RSV infection. Developing NALT in younger mice and fully formed NALT in adult mice had both PAX5-positive and -negative cells. PAX5-positive cells formed distinct aggregates that often represented organizing germinal centers, which is consistent with the role for PAX5 in B-cell lymphopoiesis^34^. PAX5-positive cells were also present in low numbers at all levels of the nasal cavity (Fig. 2h). There were no apparent differences in PAX5-positive cells in NALT after primary infection. Adult mice had an overall increase in the cellularity of lungs, which was characterized by scattered clusters of peribronchiolar inflammation and organized bronchus-associated lymphoid tissue. Compared with neonatal mice, these histologic changes were present at 7, 14, and 21 dpi. Upon reinfection, PAX5 cells in neonatal mice receiving IFN-α and in adult mice were comparable. Taken together, these findings suggest that both age and level of IFN-α play a key role in B-cell production, induction of mucosal-associated lymphoid tissue, and immune cell activation in the respiratory tract of mice after primary RSV infection or re-infection.

### IFN-α increases BAFF and APRIL expression

**Figure 3:**
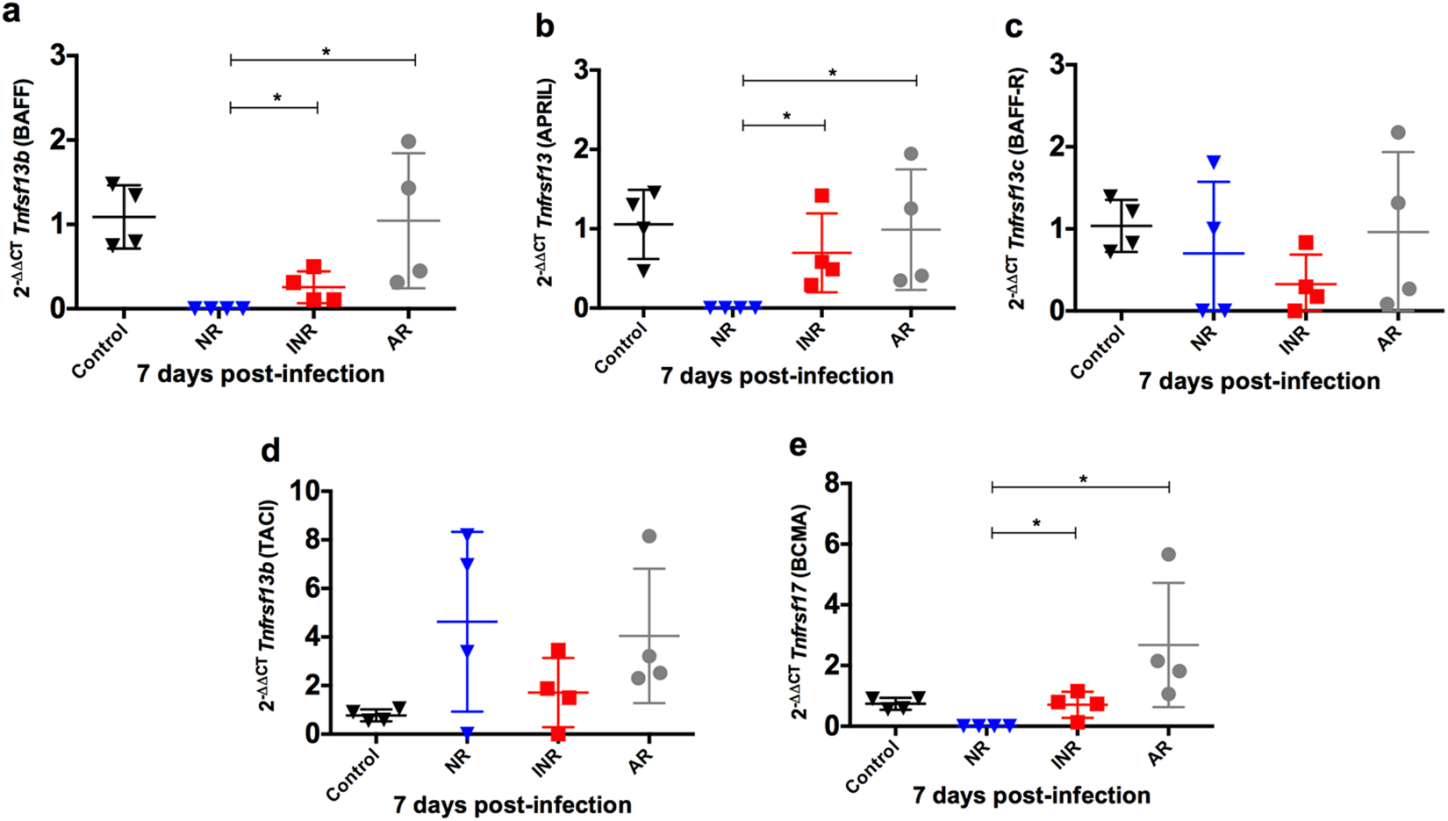
BAFF and APRIL expression is enhanced by IFN-α in NALT upon primary infection with RSV. Gene expression in NALT 7 days post infection with RSV from RSV-infected adult (AR) and neonatal mice receiving IFN-α (INR) or placebo (NR) 16 h before RSV infection. Controls were inoculated with media. (**a**) *Tnfsf13b* (BAFF); (**b**) *Tnfsf13* (APRIL); (**c**) *Tnfrsf13c* (BAFF-R); (**d**) *Tnfrsf13b* (TACI) and (**e**) *Tnfrsf17* (BCMA). n = 3–5; N = 2 *P < 0.05, **P < 0.01.

**Figure 4:**
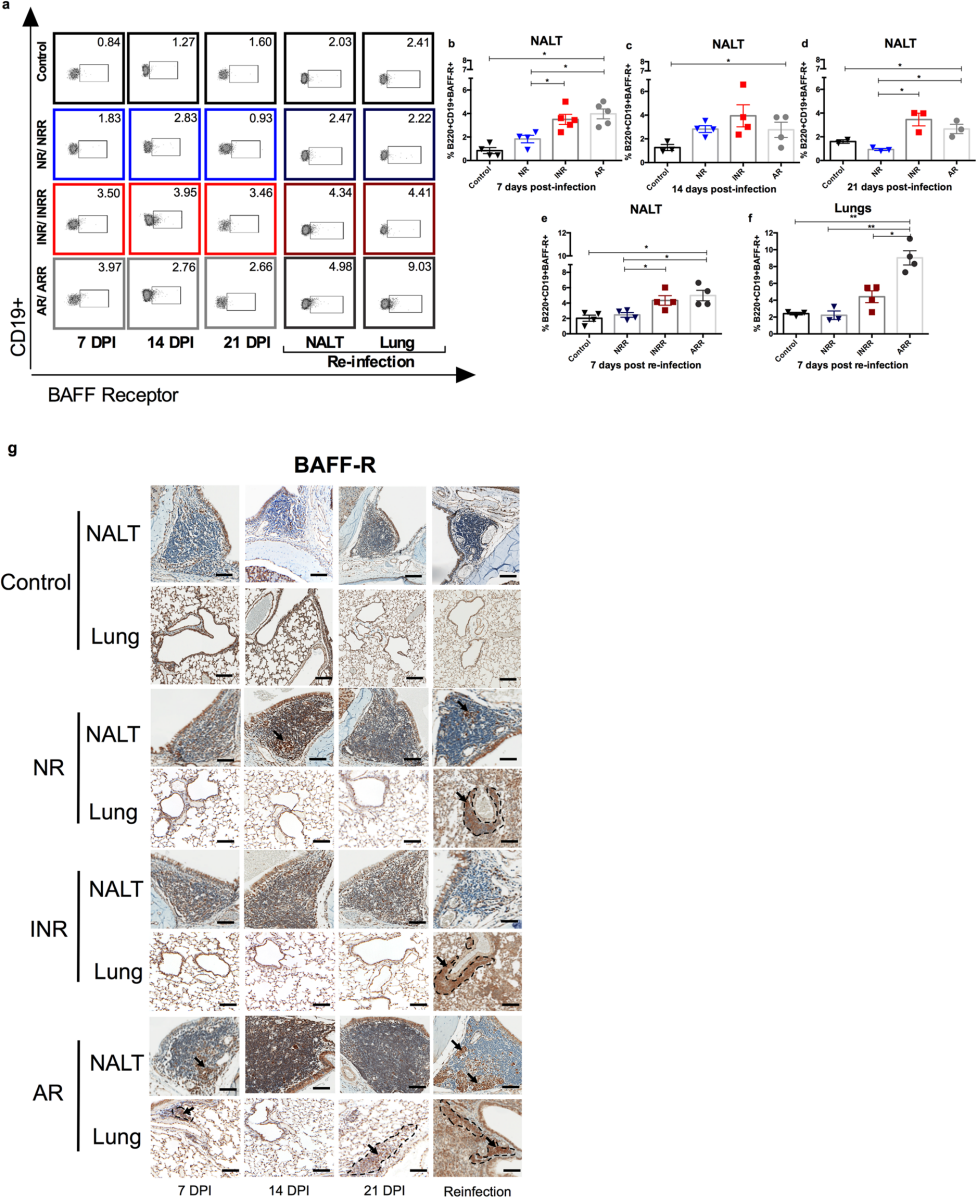
BAFF-R–positive cells are increased in NALT by IFN-α administration or age in NALT upon primary infection with RSV. BAFF-R–positive cells by flow cytometry in NALT and lungs from RSV-infected adult (AR) and neonatal mice receiving IFN-α (INR) or placebo (NR) 16 h before RSV infection and adult (ARR) and neonatal mice receiving IFN-α (INRR) or placebo (NRR) 16 h before RSV infection and reinfected 4 weeks after primary infection. (**a**) Representative flow cytometry panels of B220^+^CD19^+^BAFF-R^+^ cells. (**b–f**) Percentage of B220^+^CD19^+^BAFF-R^+^ cells at different time points. (**b**) 7 dpi; (**c**) 14 dpi; (**d**) 21 dpi; (**e**) 7 days post-secondary infection in NALT; (**f**) 7 days post-secondary infection in lungs. (**g**) IHC analysis demonstrating expression of BAFF-R protein in NALT and lungs of RSV-challenged groups and control mice at 7, 14, and, 21 days post infection, as well as, after reinfection. Magnification 200×; scale bar = 100 μm. n = 3–5; N = 2 *P < 0.05, **P < 0.01.

B-cell activating factor/tumor necrosis factor ligand superfamily member 13B (BAFF/*Tnfsf13b*) and a proliferation-inducing ligand/tumor necrosis factor ligand superfamily member 13 (APRIL/*Tnfsf13*), along with receptors tumor necrosis factor receptor superfamily member 13 C (BAFF-R/*Tnfrsf13c*), transmembrane activator and CAML interactor/tumor necrosis factor receptor superfamily member 13B (TACI/*Tnfrsf13b*), and B-cell maturation antigen/tumor necrosis factor receptor superfamily member 17 (BCMA/*Tnfrsf17*) have been implicated in T-cell–independent IgA production^35,36^. To determine the role of IFN-α in B-cell activation, expression levels of these key mediators were determined in NALT. After RSV infection, expression of BAFF and APRIL was higher in adult mice than in neonatal mice. Neonatal mice receiving IFN-α before RSV infection had higher expression of BAFF and APRIL than did neonatal mice not receiving IFN-α (Fig. 3a,b). There was no difference in the expression of BAFF-R between neonatal mice receiving or not receiving IFN-α before RSV infection (Fig. 3c). Class switching and differentiation of B cells to plasmablasts upon exposure to IFN-α/β from plasmocytoid dendritic cells (pDCs) is associated with downregulation of TACI^37^. Adult mice had lower expression of TACI than did neonatal mice and this expression seemed lower after IFN-α administration, although these differences were not significant (Fig. 3d). Interestingly, BCMA expression, which plays a role in maintaining the survival of long-lived plasma cells^38^, was significantly higher in neonatal mice receiving IFN-α and in adult mice when compared to controls and neonatal mice infected with RSV that did not receive IFN-α (Fig. 3e). When comparing mice pretreated with palivizumab with those treated with IFN-α, the former had a significant increase in BAFF expression (logFC of INR vs Palivizumab −2.276; FDR 0.006. Supplementary Table 3), further confirming that inducing IFN-α gene expression leads to increase expression of BAFF. Flow cytometry analysis for the presence of BAFF-R^+^ cells revealed that adult and neonatal mice treated with IFN-α had significantly higher numbers of CD19^+^B220^+^BAFF-R^+^ cells in NALT at 7 and 21 dpi (Fig. 4a,b,d), but there was no difference at 14 dpi (Fig. 4c). After reinfection, the response pattern in NALT and lungs was different. Neonatal mice receiving IFN-α and adult mice had a significantly higher percentage of CD19^+^B220^+^BAFF-R^+^cells in NALT, but only adult mice had an increased number of these cells in the lower respiratory tract (Fig. 4e,f). BAFF- positive cells were often concentrated in germinal centers, but were also found in other areas of NALT, with low numbers of positive cells being present at all levels of the nasal cavity in all groups. NALT of adult mice contained more BAFF-positive cells than that of neonatal mice, appearing as early as 7 dpi (Fig. 4g). BAFF-positive cells corresponded to areas of PAX5 positivity and germinal center formation in NALT in all groups, and they were predominant in adult mice at 7 dpi (Fig. 4g). Also, BAFF was expressed in both the mucosal epithelium and submucosal glands. Lungs of neonatal mice receiving IFN-α and of adult mice had comparable numbers of BAFF-positive cells at 21 dpi, and these numbers were higher than in neonatal mice not receiving IFN-α (Fig. 4g). Taken together, these data suggest a role for IFN-α in B-cell activation through BAFF/*Tnfsf13b* and APRIL/*Tnfsf13* in response to RSV infection.

### Age and IFN-α determine IgA production

**Figure 5:**
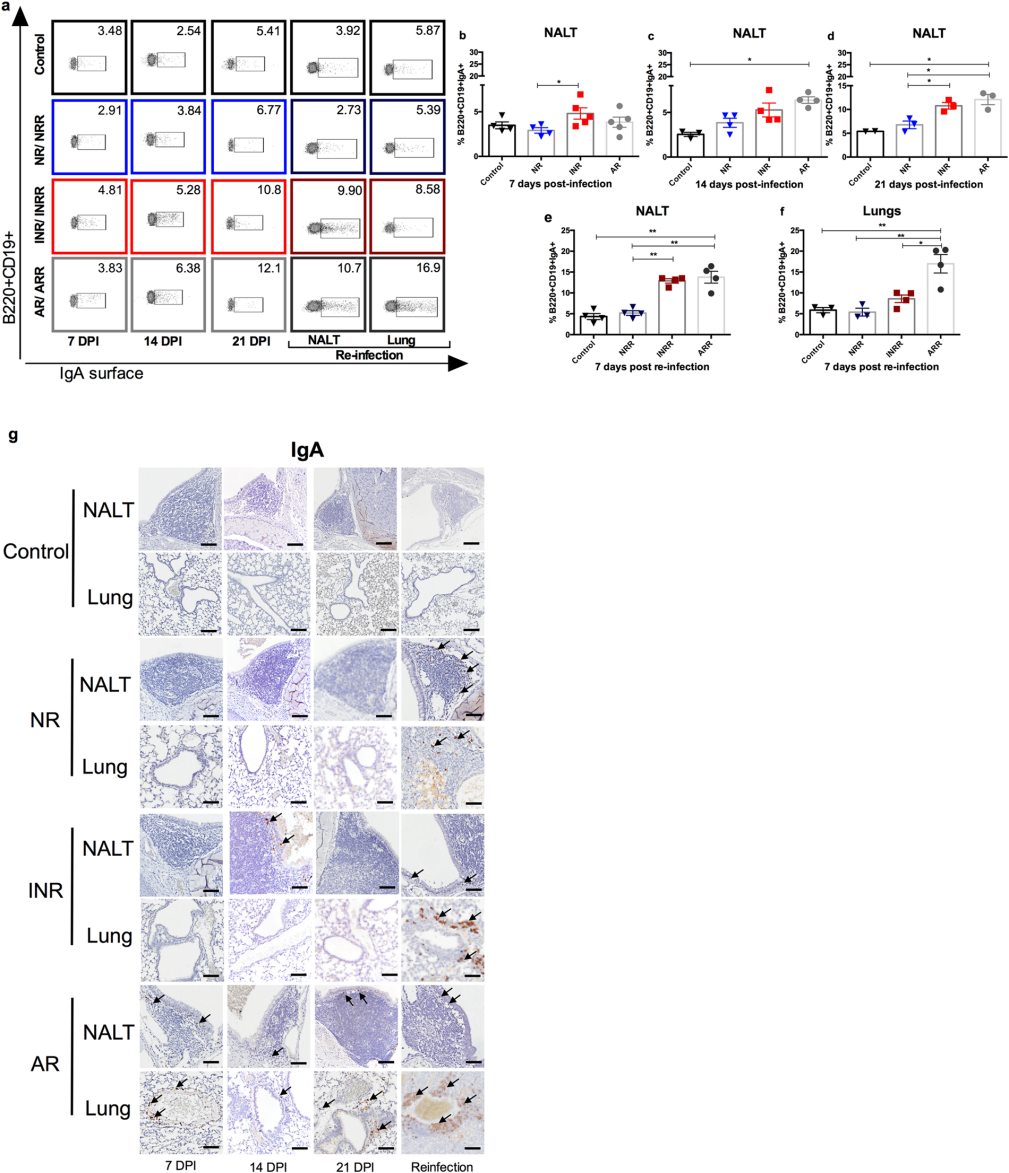
IgA-positive cells are increased in NALT by IFN-α administration or age upon primary infection with RSV. Surface IgA-positive cells by flow cytometry in NALT and lungs from RSV-infected adult (AR) and neonatal mice receiving IFN-α (INR) or placebo (NR) 16 h before RSV infection and adult (ARR) and neonatal mice receiving IFN-α (INRR) or placebo (NRR) 16 h before RSV infection and reinfected 4 weeks after primary infection. (**a**) Representative flow cytometry panels of B220^+^CD19^+^IgA^+^ cells; (**b–f**) Percentage of B220^+^CD19^+^IgA^+^ cells at different time points. (**b**) 7 dpi; (**c**) 14 dpi; (**d**) 21 dpi; (**e**) 7 days post-secondary infection in NALT; and (**f**) 7 days post-secondary infection in lungs. (**g**) IHC analysis demonstrating expression of IgA protein in NALT and lungs of RSV-challenged groups and control mice at 7, 14, and, 21 days post infection, as well as after reinfection. n = 3–5; N = 2 Magnification 200×; scale bar = 100 μm.

To determine the role of age and IFN-α in IgA production upon RSV infection, NALT of mice was harvested at different time points after primary infection. B cells expressing surface IgA were measured by flow cytometry. Mice that received IFN-α had significantly higher proportions of IgA-expressing B cells at 7 dpi (Fig. 5a,b). At 14 dpi all infected mice had higher numbers of IgA-expressing B cells than did uninfected mice (Fig. 5c). At 21 dpi, neonatal mice receiving IFN-α and adult mice had significantly higher numbers of IgA-expressing B cells than did neonatal mice and controls (Fig. 5d). Interestingly, upon reinfection, this difference was notable in NALT as early as 7 dpi (Fig. 5e). IgA-expressing B cells in lungs were significantly increased in adult mice and slightly elevated in mice receiving IFN-α, but the latter increase was not significant (Fig. 5f). Similar results were observed upon reinfection when intracellular IgA was measured (Supplementary Fig. 4a–c). IgA-positive cells were present within and around NALT and at all histologic levels of the nasal cavity. The maximum increase in IgA-positive cells occurred in adult mice at 7, 14, and 21 dpi and to a lesser extent in neonatal mice receiving IFN-α at 21 dpi compared with controls and neonatal mice not receiving IFN-α (Fig. 5g). IgA-positive cells correlated with fully developed NALT, IFN-α administration before RSV infection, increased age of mice at the time of viral challenge, and secondary infection to RSV.

IgA-positive cells were observed in lungs of adult mice adjacent to areas of inflammation and correlated with IgA^+^ secretions. Interestingly, IFN-α administration did not increase IgA in lungs upon primary infection, but correlated with an increase in IgA-positive cells in lungs upon reinfection (Fig. 5g).

**Figure 6:**
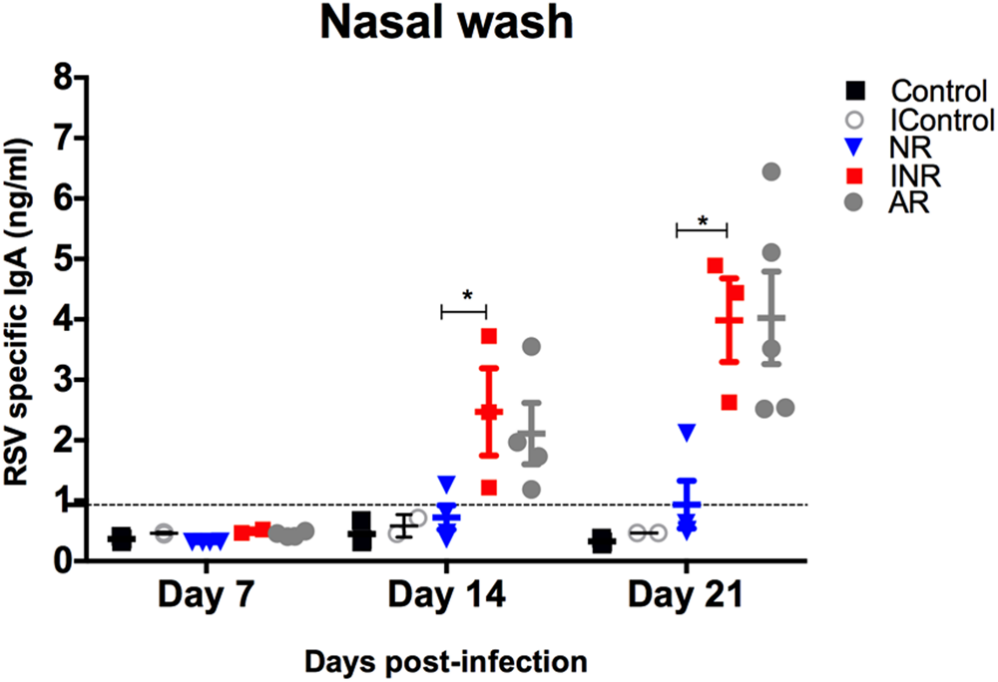
RSV-specific mucosal IgA in the upper respiratory tract is increased by IFN-α administration before RSV infection. RSV-specific mucosal IgA in nasal washes at different time points from RSV-infected adult (AR) and neonatal mice receiving IFN-α (INR) or placebo (NR) 16 h before RSV infection. Controls received vehicle control before RSV infection. A subset of neonatal mice received IFN-α followed by media (IControl). Dotted line represents lower limit of detection of the assay. n = 3–5; N = 2.

Lastly, RSV-specific IgA levels were measured in nasal wash, NALT, BAL, and lung homogenates (Fig. 6 and Supplementary Fig. 5a–c). As expected, RSV-specific IgA in nasal washes was comparable between neonatal mice receiving IFN-α and adult mice at 14 dpi (Fig. 6). Taken together, these findings suggest that the effect of age in IgA production upon RSV infection can be overcome by IFN-α administration.

## Discussion

Our study highlights age-related differences in mucosal immune response against RSV infection in mice. IFN-α administration before RSV infection in neonatal mice induced BAFF and APRIL expression at levels comparable with those in adult mice. Furthermore, IFN-α augmented the production of total IgA producing B cells and RSV-specific IgA levels in NALT and reduced inflammation upon reinfection.

We describe a dual role for IFN-α as an antiviral and immune modulator that goes beyond primary RSV infection. Early reduction of viral burden in the respiratory tract can have short and long term immune effects^39^. A previous study showed that when type I IFN pathways are reconditioned in neonatal mice upon RSV infection, DC numbers in the lungs increase and mice are protected from exacerbated airway disease upon RSV reinfection in adulthood^24^. Also, this is accompanied by a shift toward a Th1 response. Adult mice produce significantly higher levels of type I IFN in response to RSV infection than do neonatal mice^23^. Furthermore, IFN-α administration or passive transfer of adult pDCs (which can produce type I IFN) before RSV infection protects mice against airway hyper-reactivity and decreased pulmonary Th2 bias^23^. These results agree with our current findings that neonatal mice receiving IFN-α had the lowest viral load in nasal washes and less inflammation upon reinfection. Furthermore, gene expression analysis of mice treated with Palivizumab, which decreases infectivity and viral load in mice^40,41^, suggests that while some of the effects shown in Fig. 1 are indeed due to an attenuated infection, there is a distinct role for IFN-α administration on the mucosal immune response against RSV infection. Tripp *et al*. have recently reported that treatment with TRL3D3 (a monoclonal antibody targeting G) lead to enhanced IFN whereas anti-F protein Mab depressed the IFN response^42^. These mice had decreased airway inflammation, and improved lung function upon secondary infection, whereas mice treatead with anti-F had less IFN than mock infected animals^42^. Capella *et al*. showed that elevated titer to G was as well correlated with mild diseaseas as was titer to pre-F in infants with RSV infections suggesting that targeting G has an important role in clinical outcome^43^. These results confirm the role of IFN-α as an antiviral and an immune modulator upon RSV infection and supports our hypothesis that IFN is critical in the development of the mucosal immune response against RSV and that those effects can not be fully explained by attenuation of the infection.

We did not observe any differences in gene expression associated with B cell or BCR signaling when comparing neonates with and without previous IFN-α administration. Differences in expression are for the entire lung and not specific to cell populations; thus the actual expression changes in specific cells may misrepresent expression in B cells or cells producing BAFF and TACI such as pDCs. When comparing mice treated with IFN with those who were B cell depleted, there are different GO terms related to BCR signaling. Interestingly, mice depleted of B cells had significantly higher viral load in NALT and lungs. B cell depletion has been shown to impair CD4+ T cell activation and clonal expansion in response to protein antigens and pathogen challenge. Furthermore, CD20 immunotherapy (such as the one we have used in our experiments) revealed that optimal antigen-specific CD4+ T cell priming requires B cells, and hence the difficulty in controlling viral replication^44^.

There was a discrete but significant increase in B-cell numbers in NALT of neonatal mice receiving IFN-α at 14 dpi compared with uninfected controls. This can be explained by increased expression of BAFF and APRIL earlier in infection in both neonatal mice receiving IFN-α and adult mice. Further, we showed that expression of BAFF and APRIL after RSV infection differed by age (lower in neonatal mice than mature mice), and this difference was overcome by IFN-α administration. Besides being essential for B-cell expansion and survival^45^, BAFF is implicated in regulating T cell–independent antibody production^46^. BAFF has been localized in the infected respiratory epithelium of lungs from infants with fatal bronchiolitis^47^, and human airway epithelial cells produce BAFF in response to RSV infection *in vitro*^48^. However, this may not be sufficient to induce an immune memory response. There are well-defined age-related differences in the BAFF/BAFF-R pathway. BAFF-R expression is low in newborns, and this is associated with decreased B-cell survival^49^. When B cells from human preterm neonates and adults are stimulated with recombinant BAFF, B cells from preterm neonates have less proliferation and lower expression of BAFF-R than those from adults. In fact, BAFF or APRIL cannot induce immunoglobulin secretion from neonatal B cells *in vitro*^49^. These observations can be explained by the lack of type I IFN production at young ages.

High titers of circulating antibodies against RSV in the blood can protect against lower respiratory tract infections^50,51^. However, there is increasing interest in mucosal antibodies from nasal washes, as they are localized to the first point of contact with RSV^28,29,52^. Vissers *et al*. showed that mucosal IgG had a higher correlation with RSV viral load and inflammation than did systemic IgG^30^. In two experimental human models of RSV, prior RSV-specific nasal IgA correlated significantly more strongly with protection from infection than did serum neutralizing antibodies^28,29^. Interestingly, one of these studies showed that only IgG levels remained high in the convalescence period, indicating the presence of a potential mechanism that explains multiple infections by RSV despite its relative antigenic stability^28^. We are currently exploring this aspect in our mouse model.

In conclusion, we showed an age-related difference in IgA production against RSV infection. Neonatal mice did not produce IgA in both the upper and lower respiratory tracts in response to RSV. This age-related difference in the immune response could be explained by lack of type I IFN signaling in neonatal mice, which in turn reduces B-cell activation and IgA class switching. With approximately 60 RSV vaccine candidates currently under development, insights into the mucosal immune response against RSV infection and its age-related differences are critical to identify and guide the development of effective immunologic adjuvants for vaccines, understand mechanisms underlying RSV reinfection and severity, and emphasize the need to consider differential interactions between virus and host immune response at different ages.

## Methods

### Mice

BALB/c mice (Jackson Laboratories) were maintained in specific-pathogen-free facilities at the University of Tennessee Health Science Center (UTHSC), Memphis, TN. Mice to be bred were time mated, and pups born on the same date were used for experiments. **NR**, neonatal mice infected with RSV; **INR**, neonatal mice receiving IFN-α 16 h before RSV infection; **AR**, adult mice infected with RSV; **NRR**, adult mice infected with RSV as neonates and reinfected 4 weeks later; **INRR**, adult mice infected with RSV receiving IFN-α 16 h before RSV infection as neonates and reinfected 4 weeks later; **ARR**, adult mice infected with RSV twice at a gap of 4 weeks (Supplementary Fig. 6). N = replicate experiments with n animals per group per experiment. All animal protocols were performed in accordance with the Guide for the Care and Use of Laboratory Animals and approved by the Institutional Animal Care and Use Committee at UTHSC.

### IFN-α treatment

Mouse recombinant IFN-α A (12105-1) was purchased from PBL Interferon Source (Piscataway, NJ). Sixteen hours before RSV infection, 2 × 10^3^ units (dose extrapolated from that used in adultmice)^53^ of IFN-α was intranasally instilled into pups in 10 μL of PBS containing 0.1% bovine serum albumin (BSA). Control pups (NS) received 10 μL of PBS containing 0.1% BSA.

### B-cell depletion

Purified anti-mouse CD20 antibody was purchased from BioLegend. Sixteen hours before RSV infection, 250 µg of Ultra-LEAF™ purified mAb anti-mouse CD20 was intraperitoneally injected into pups. In addition, 10 µg was intranasally instilled.

### Palivizumab treatment

After reconstitution from lyophilized powder in 100 μl of sterile water, a dose of 50 mg/kg (1.25 mg per mouse) was administered once intraperitoneally, 24 h before intranasal inoculation, as described elsewhere^40,41^.

### RSV infection

Human RSV strain A2 (Advanced Biotechnologies) was propagated in Vero cells (ATCC) grown in HyClone™ serum-free media (ThermoFisher Scientific), harvested using a standard protocol^23^, and stored at –80 °C until use. Mice (5-day-old pups or 6- to 8-week-old adults) were intranasally infected with RSV in serum-free media at a dose of 2 × 10^5^ tissue culture infectious dose 50 per gram of body weight. Control mice received serum-free media.

### NALT dissection

NALT tissues were collected as previously described^54^. Briefly, euthanized mice were decapitated. After the lower jaw, including tongue, was removed, palates were scored by cutting along the inside edges of the upper molars with a scalpel and then peeling the tissue away from the roof of the mouth. Once exposed the entire hard palate and NALT were excised.

### Real-time PCR

RNA was isolated from frozen nasal-associated lymphoid tissue (NALT) and lungs by using the RNeasy Plus Mini Kit (Qiagen) as per the manufacturer’s instructions. Real time RT-PCR was performed using the SuperScript™ III Platinum™ One-Step qRT-PCR Kit (Life Technologies).

Simple relative quantification of target gene expression normalized to *Hprt* was performed using the 2^−ΔΔCt^ method as described elsewhere^55^. Control group changed depending on the experiment. Control groups for Fig. 1b was NR (neonatal mice infected with RSV); for Fig. 3 was uninfected neonate; and for Supplementary Fig. 2f was NR.

The following primers were used: NS1, forward primer: 5′-CACAACAATGCCAGTGCTACAA-3′; NS1, reverse primer: 5′-TTAGACCATTAGGTTGAGAGCAATGT-3′; *Hprt* forward primer: 5′-GGCTCCGTTATGGCGACCCG-3′; *Hprt* reverse primer: 5′-CGAGCAAGACGTTCAGTCCTGTCC-3′. TaqMan™ primers and probes for *Tnfsf13b*, *Tnfrsf13c*, *Tnfrsf13b*, *Tnfsf13*, *Tnfrsf17*, and *Ifn-*α4 were purchased from Applied Biosystems.

### Flow cytometry

Single cells were isolated and labeled with a fixable viability dye and antibodies to IgA-FITC (BD Biosciences, #553478); IgA-PE (BD Biosciences, #562141); CD69-BV786 (BD Biosciences, #56468); B220-BV605 (BD Biosciences, #563708); CD19-PerCp-Cy5.5 (BD Biosciences, #551001); CD11c-PE-CY7 (BD Biosciences, #558079); CD317 (pDCA1)-BV421 (BD Biosciences, #566431); CD268 (BAFF Receptor)-APC (eBioscience, #17-9117-42); and LIVE/DEAD™ Near IR- Dead Cell Stain Kit (Molecular Probes, #L34975). Isotype controls were used to set the photomultiplier tube (PMT) voltages. Single color controls were used for compensation. Stained cells were assayed on the LSRFortessa™ cell analyzer (BD Biosciences), and flow data were analyzed and plotted using BD FACSDiva™ (ver. 8). Singlet live cells were gated before further analysis (Supplementary Fig. 1).

### RSV specific IgA ELISA

Nunc-Immuno MaxiSorp™ plates were coated with RSV (10^5^ plaque-forming units/mL) overnight. Test samples were diluted and incubated in microtiter wells for 45 min alongside mouse IgA standards, using IgA ELISA (Life Diagnostics, #IGA-1). Horseradish peroxidase conjugate was added and incubated for 45 min, followed by incubation with TMB reagent for 20 min at room temperature. Color development was stopped by adding the stop solution, and optical density was spectrophotometrically measured at 450 nm. The IgA concentration was derived from a standard curve with a range of 0.93–30 ng/mL.

### Pathology analysis

Mice heads and lungs were harvested and fixed with 10% neutral buffered formalin for 72 h before standard histologic processing, which included decalcification of bony tissues, sectioning, and hematoxylin-and-eosin (H&E) staining. Immunohistochemistry (IHC)-based labeling was performed using a Discovery XT autostainer (Ventana Medical Systems) or a BOND RX autostainer (Leica Biosystems). Primary antibodies used to label serial sections of lungs and NALT were anti-BAFF antibody (BAFF, clone T7-241, 1:100 concentration, MyBioSource, #MBS241873), anti-IgA antibody (IgA, 1:16,000 concentration, Bethyl Laboratories, #A90-104A) or anti-IgG antibody (IgG, 1:1500 concentration, Novus Biologicals, #NB7588), using a Ventana Discovery XT autostainer. Serial sections of lungs or NALT were labeled with an anti-PAX5 antibody (PAX5, 1:125 concentration, Abcam, #ab109443), using a Leica Bond RX autostainer. All slides were counterstained with hematoxylin. At least two H&E sections for each tissue and IHC-labeled serial sections were analyzed in a blinded manner by a board-certified veterinary pathologist (HT). Bright-field images were taken with an upright Eclipse Ni (Nikon) or constructed from digitized images using Aperio ImageScope (Leica Biosystems).

To quantify the extent of inflammation in tissue sections, H&E–stained sections of the lung tissue were digitized to 20× scalable images using Aperio ScanScope (Leica Biosystems). Static 1× images were generated by ImageScope (Leica Biosystems). Images were classified as lung tissue, immune cell infiltrates, other tissues, blood, and slide glass by using Fiji. The percentage of inflammation within the lungs was determined by dividing pixels classified as immune cell infiltrates by pixels classified as lung tissue. Results were graphed using GraphPad Prism v6.0.

### Gene expression analysis

RNA was isolated from frozen NALT and lungs using the RNeasy Plus Mini Kit (Qiagen) as per the manufacturer’s instructions. Samples were assayed using Clariom™ S mouse HT arrays (Affymetrix). Robust multi-array average normalization and statistical testing to determine differential expression was performed with Partek Genomics Suite 6.6. Category enrichment was tested using Enrichr^56^, and pathway analyses were performed using Ingenuity^®^ Pathway Analysis (Qiagen). Filtering, deduplication, and visualizations such as scatterplots and volcano plots were performed using STATA 14.2/MP. Principal component analysis (PCA) was performed, and heatmaps were generated using Partek Genomics Suite 6.6^56,57^. P values post adjustment for false discovery rate.

The datasets generated during and/or analysed during the current study are available from the corresponding author on reasonable request.

### Statistics

Data were plotted as means ± standard errors (SEM) and analyzed using Prism 6 (GraphPad Software; La Jolla, CA, USA). Two-way analysis of variance (ANOVA) and student’s t-test was used for all mouse studies. Each figure represents one experiment. In the legend, n represents the number of animals and every experiment was repeated at least twice. Differences were considered significant if p < 0.05.

## Electronic supplementary material

Supplementary materials

Dataset 1

Dataset 2

Dataset 3

Dataset 4
