# Supplementary material for: Type I Interferon Potentiates IgA Immunity to Respiratory Syncytial Virus Infection During Infancy
Source: Sci Rep. 2018 Jul 23;8:11034. doi: 10.1038/s41598-018-29456-w (PMC6056463; doi:10.1038/s41598-018-29456-w)
Supplement: Supplementary file 1 — Supplementary materials [file 41598_2018_29456_MOESM1_ESM.pdf]

"Type I interferon potentiates humoral immunity in the nasal mucosa": Supplementary material

Diego R. Hijano[1], David T. Siefker[2], Bishwas Shrestha[2], Sridhar Jaligama[2], Luan D Vu[2], Heather Tillman[3], David Finkelestein[4], Jordy Saravia[2], Dahui You[2], and Stephania A. Cormier[2,5,*]

[1]Department of Infectious Diseases, St. Jude Children's Research Hospital, Memphis, Tennessee

[2]Department of Pediatrics, University of Tennessee Health Science Center, Memphis, Tennessee

[3]Department of Pathology, St. Jude Children's Research Hospital, Memphis, Tennessee

[4]Department of Computational Biology, St. Jude Children's Research Hospital, Memphis, Tennessee

[5]Children's Foundation Research Institute, Memphis, Tennessee

Supplementary Figure 1: Gating strategy

Supplementary Figure 2 (related to Figure 1):

(A) Venn diagram for the numbers of common and specific genes in NALT of neonatal mice that received IFN-α (INR) compared to those that received media (NR) or were depleted of B cells prior to RSV infection. (B) Description of GSEA analysis plot. (C) GSEA comparison for INR and NR NALT for granulocyte migration, neutrophil immunity, regulation of T cell proliferation and MAPK activity. (D) Volcano plot showing significance versus fold change of gene expression of neonatal mice that received IFN-α (INR) vs neonatal mice that received palivizumab. (E) Principal component analysis of lung gene expression of neonatal mice that received IFN-α (INR) compared to those that received media (NR) or were depleted of B cells prior to RSV infection. (F) *NS1* gene expression in lungs from RSV-infected adult (gray) and neonatal mice receiving IFN-α (IRN/red), placebo (NR/blue), or anti CD-20 (green) 16 h before infection. n=3-5; N=2 $^*P < 0.05$, $^{**}P < 0.01$

Supplementary Figure 3 (related to Figure 1):

Percentage of inflammation in lungs from RSV-infected neonatal mice receiving IFN-α (INNR/maroon) or placebo (INR/blue) 16 h before infection. Controls were inoculated with media (black). n=3-5; N=2 $^*P < 0.05$, $^{**}P < 0.01$

Supplementary Figure 4 (related to Figure 5):

Intracellular IgA positive cells by flow cytometry in NALT and lungs from adult (ARR) and neonatal mice that received IFN- α (INRR) or placebo (NRR) 24 hrs. prior to RSV infection, reinfected 4 weeks after primary infection (A-C). Intracellular IgA. (A) Representative flow cytometry panels of B220+CD19+IgA+ cells. (B) Percentage of B220+CD19+IgA+ 7 days post-secondary infection in NALT; (C) Percentage of B220+CD19+IgA+ 7 days post-secondary infection in lung. n=3-5; N=2

Supplementary Figure 5 (related to Figure 6):
RSV specific mucosal IgA in the respiratory tract.
(A-C) RSV specific mucosal IgA at 21 days post-infection different time points from RSV infected adult (AR) and neonatal mice (NR). Age matched controls for adult (AS) and neonatal (NS) mice. (A) NALT; (B) Bronchoalveolar lavage; (C) Lungs homogenates. Dotted line represents lower limit of detection of the assay. n=3-5; N=2

Supplementary Figure 6:
Animal model, animal groups and different interventions used for the experiment described in the manuscript.

Supplementary Figure 1

Gating strategy

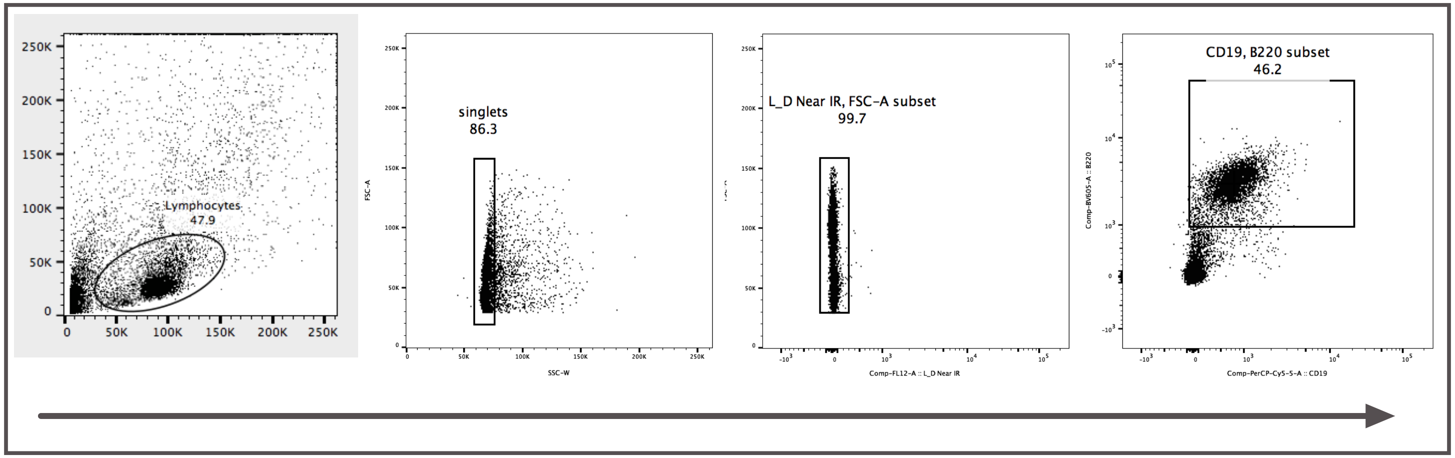

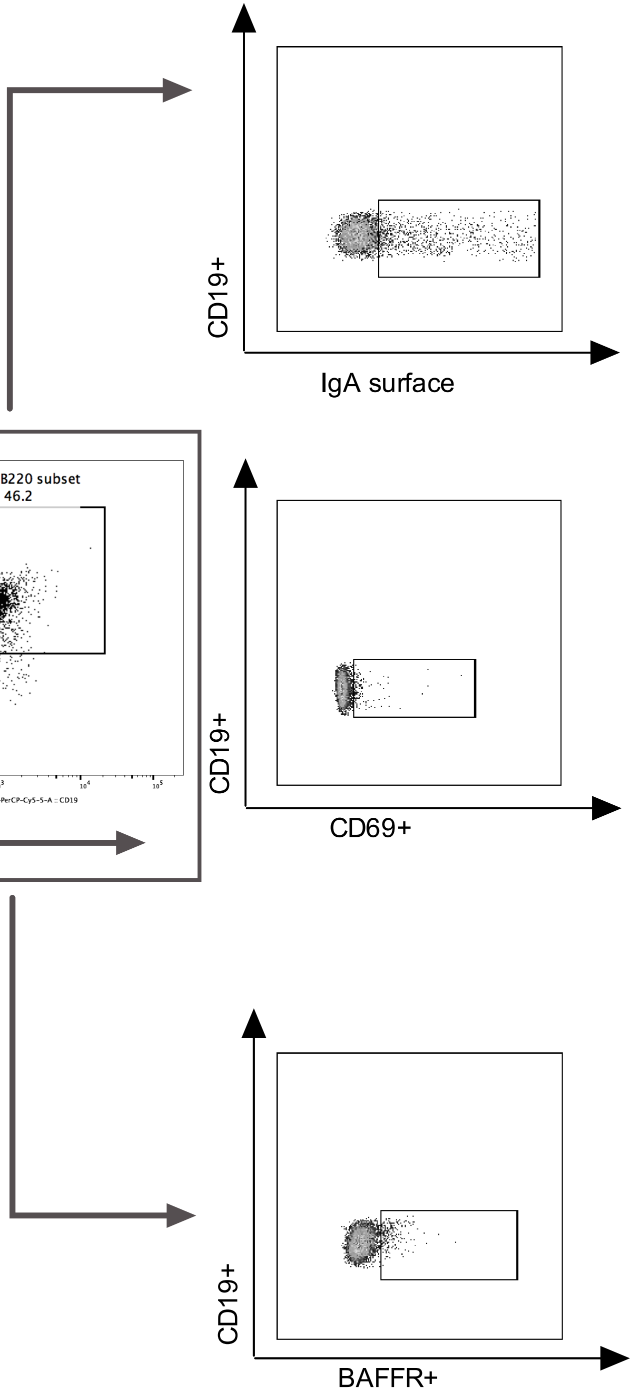

Antibodies

IgA-FITC – BD Biosciences – 553478
IgA-PE – BD Biosciences – 562141
CD69-BV786 - BD Biosciences 564683
B220-BV605 – BD Biosciences 563708
CD19-PerCp-Cy5.5 – BD Biosciences 551001
CD11c-PE-CY7 – BD Biosciences 558079
CD317 (pDCA1)-BV421 – BD Biosciences 566431
CD268 (BAFF Receptor)-APC – eBiosciences 17-9117-42
Live/Dead Near IR- Molecular Probes – L34975

Supplementary Figure 2

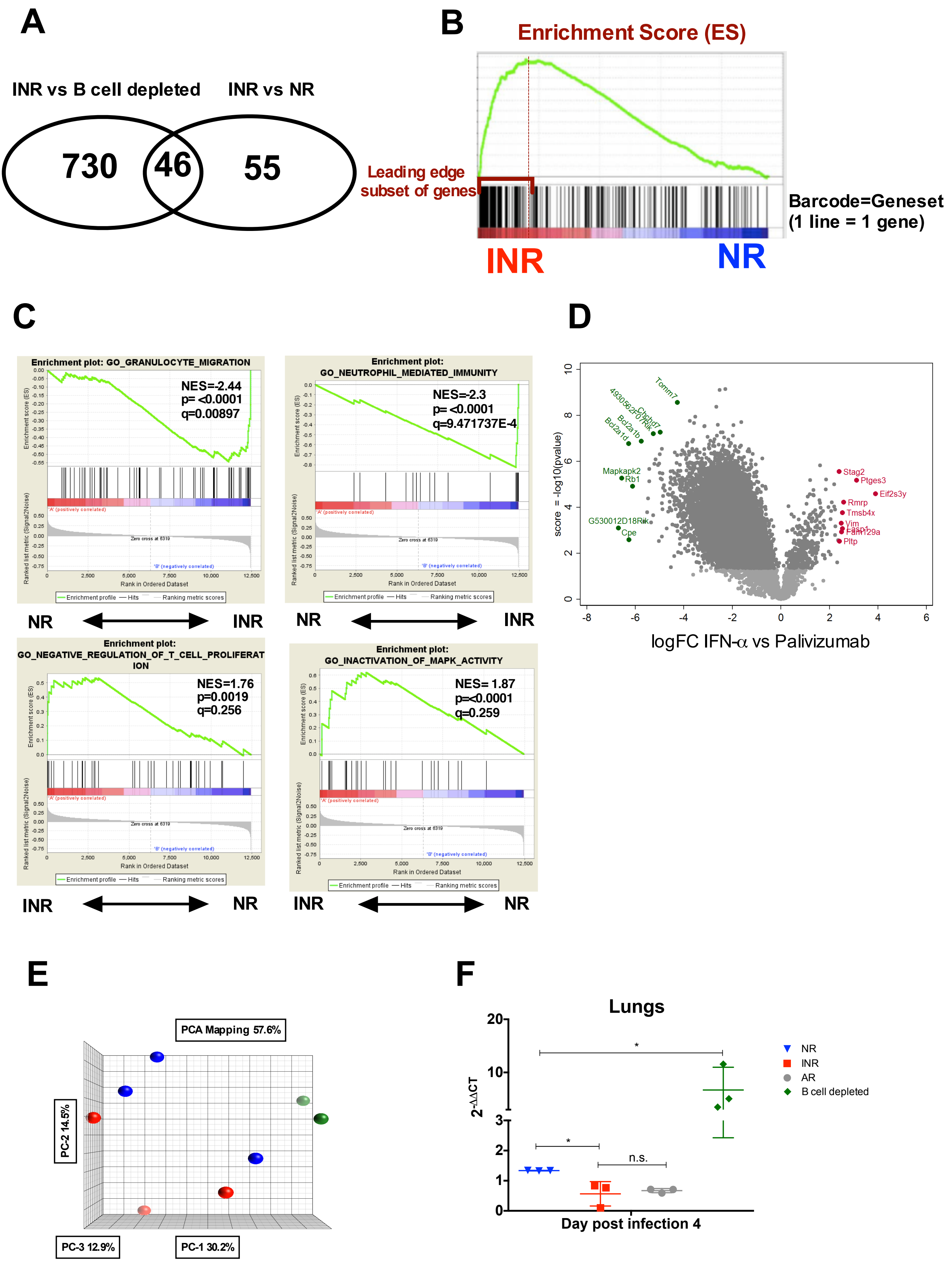

Supplementary Figure 3

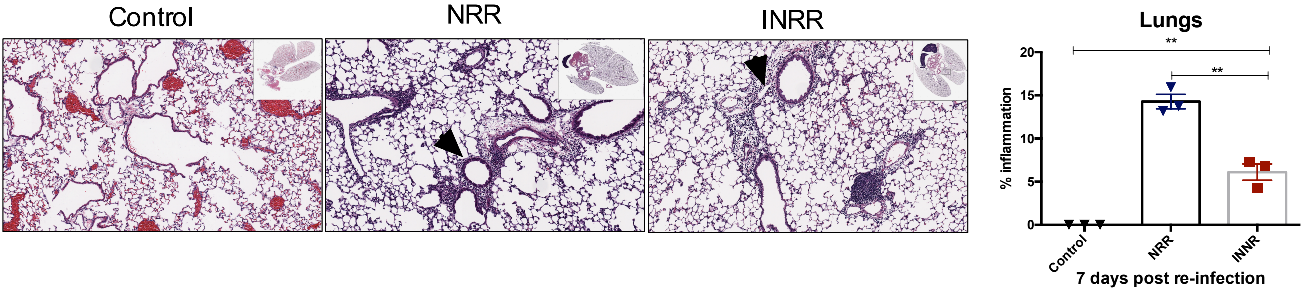

Supplementary Figure 4

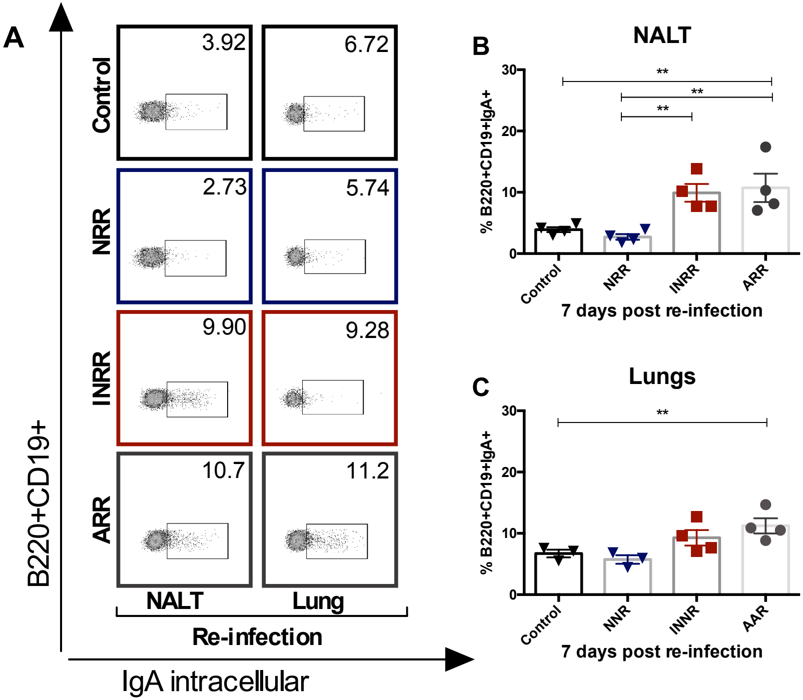

Supplementary Figure 5

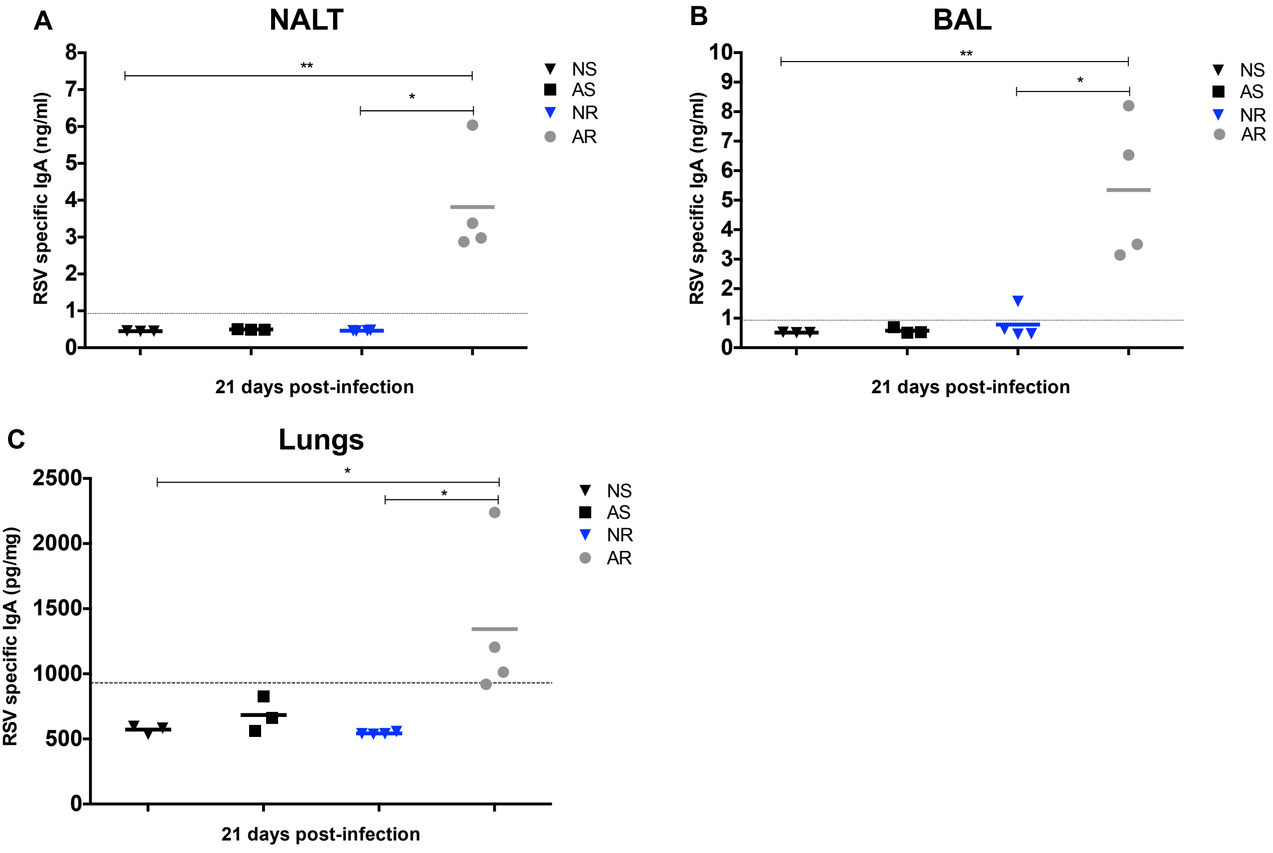

Supplementary Figure 6

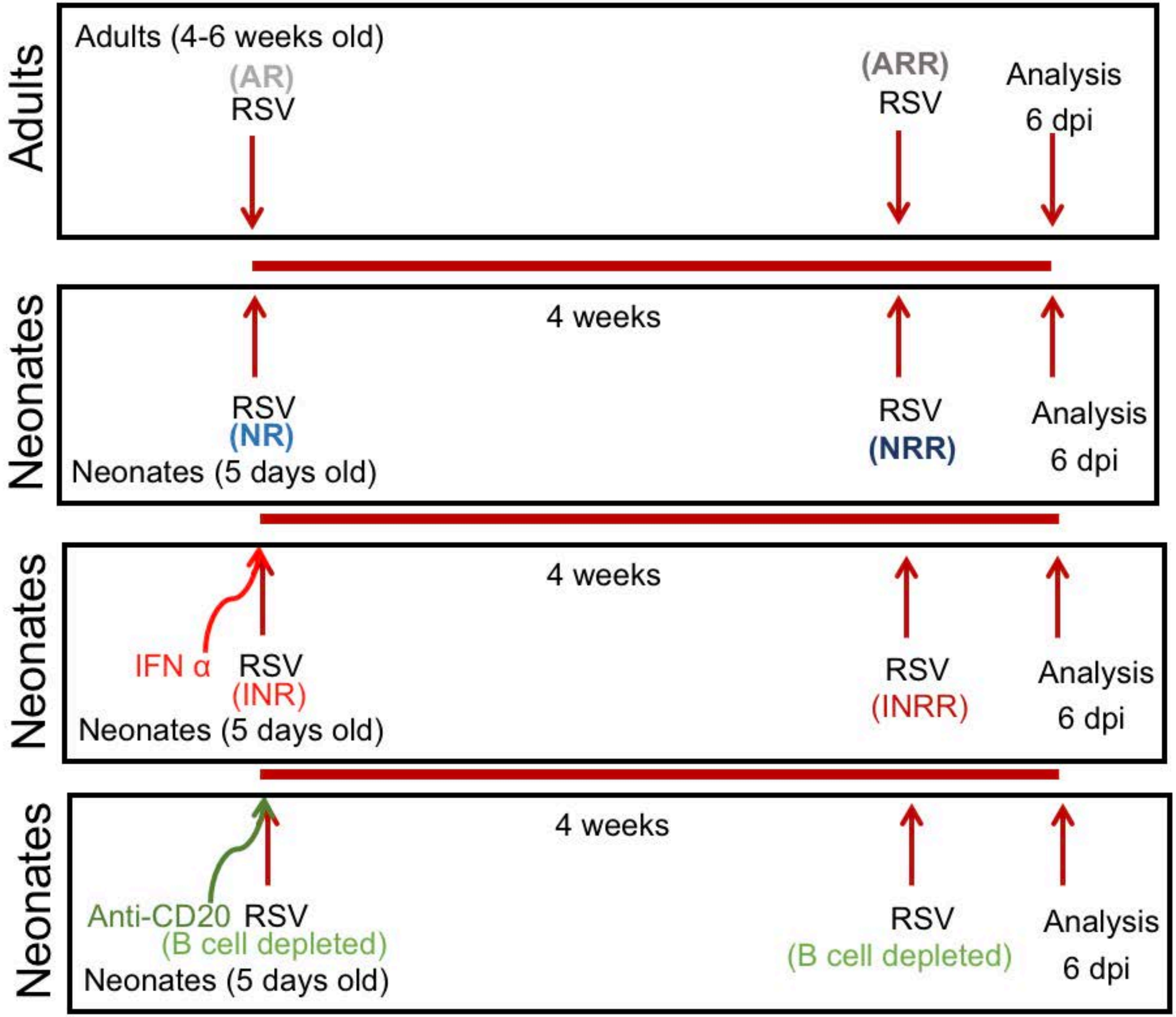

<u>Supplementary Table 1</u>: Expression of genes involved in regulation of the inflammatory response.

Gene Symbol	INR (mean)*	Palivizumab (mean)*	p value	Log FC	FDR
Xcl1	4.001560211	6.04968977	0.0255268	-2.0481296	0.03022062
Clec4g	3.353630066	5.893539906	0.00045433	-2.5399098	0.00138147
Dusp18	4.388689995	6.587800026	0.00311915	-2.19911	0.00520468
Dusp10	7.815410137	10.32989979	0.00464768	-2.5144897	0.00706941
Dusp9	3.607120037	6.599470139	0.00010193	-2.9923501	0.00060953
Dusp7	6.849430084	9.331230164	0.00035158	-2.4818001	0.00118886
Lax1	4.186950207	6.705649853	0.00328686	-2.5186996	0.00540932

*Results are expressed as means of 4 mice per group.

<u>Supplementary Table 2</u>: Expression of genes involved in granulocyte and neutrophil migration.

Gene Symbol	INR (mean)*	Palivizumab (mean)*	p value	Log FC	FDR
**Cklf**	**6.052740097**	**9.923999786**	3.6649E-05	-3.8712597	0.00038382
**Pde4b**	**7.904960155**	**10.0934**	0.00021007	-2.1884398	0.00088198
**C5ar1**	**3.90557003**	**6.657660007**	4.1685E-07	-2.75209	0.00012179
**Trem1**	**3.987540007**	**6.51928997**	0.00040153	-2.53175	0.0012811
**Edn2**	**3.812589884**	**5.981460094**	4.1943E-05	-2.1688702	0.00040904
**Il1b**	**4.342830181**	**7.820879936**	5.0005E-06	-3.4780498	0.00019068
**S100a8**	**7.316649914**	**10.30179977**	0.00582763	-2.9851499	0.00850084
**Csf3r**	**5.02891016**	**7.486780167**	0.00026783	-2.45787	0.00101522
**Ccl20**	**4.560780048**	**6.678880215**	0.00780663	-2.1181002	0.01078206
**Cxcl5**	**3.230870008**	**5.669290066**	5.2339E-05	-2.4384201	0.00044859

*Results are expressed as means of 4 mice per group.

Supplementary Table 3: IFN-α and Tnfsf13b (BAFF) gene expression.

Gene Symbol	INR (mean)*	Palivizumab (mean)*	p value	Log FC	FDR
**Ifna1**	**4.18671989**	**6.765270233**	**2.2696E-05**	**-2.5785503**	**0.00031633**
**Ifna2**	**3.32118011**	**6.461239815**	**0.00013454**	**-3.1400597**	**0.00070665**
**Ifna5**	**5.24568987**	**7.683740139**	**1.7437E-05**	**-2.4380503**	**0.00028263**
**Ifna6**	**3.72046995**	**6.45333004**	**0.00010143**	**-2.7328601**	**0.00060952**
**Ifna7**	**4.06274986**	**7.142149925**	**8.8788E-05**	**-3.0794001**	**0.00057018**
**Ifna11**	**2.98958993**	**5.263639927**	**0.00053228**	**-2.27405**	**0.00153147**
**Ifna14**	**3.24411988**	**5.841479778**	**4.226E-06**	**-2.5973599**	**0.00018788**
**Ifna16**	**3.52806997**	**6.228750229**	**3.9546E-05**	**-2.7006803**	**0.00039934**
**Tnfsf13b**	**4.44101**	**6.717189789**	**0.00376091**	**-2.2761798**	**0.00600049**

*Results are expressed as means of 4 mice per group.
